# Transmission Characteristics and Structural Optimization of Lightguide Rods for Optical Partial Discharge Detection

**DOI:** 10.3390/s26216994

**Published:** 2026-11-05

**Authors:** Chao Pan, Yong Qian, Zehao Chen, Xiaoxin Chen, Jiuyi Zhao, Gehao Sheng

**Affiliations:** 1 School of Electrical Engineering, Shanghai Jiao Tong University, Shanghai 200240, China; 2 Electric Power Research Institute, State Grid Zhejiang Electric Power Co., Ltd., Hangzhou 310014, China; 3 Hangzhou Power Supply Company, State Grid Zhejiang Electric Power Co., Ltd., Hangzhou 310016, China

**Keywords:** lightguide rod, optical detection, partial discharge, radiant flux, rear-section design

## Abstract

When a lightguide rod extracts partial discharge (PD) light from gas-insulated switchgear (GIS), losses introduced by the rear-section fixture and aperture mismatch with the fiber entrance limit the usable optical output. Ray tracing was used to examine how a parabolic taper affects optical transmission and reception through a finite aperture. The selected design increased the mean radiant flux received at the fiber entrance to 7.26 times the baseline value. In integrating sphere tests under uniform illumination, the sample with a parabolic taper provided a higher detector output than the other three samples under all tested conditions. In needle–plate corona tests in a laboratory GIS model, the main optical responses corresponded in time to the ultrahigh-frequency (UHF) envelope and shared the same temporal features. The optical channel also recorded clusters of low-amplitude responses during the negative half-cycle that were not clearly resolved by the UHF channel; these responses were accompanied by activity in the auxiliary ultrasonic channel. These results identify rear-section transmission loss and finite-aperture reception as constraints on lightguide design and provide experimental evidence for coupling lightguide rods to transmission fibers in optical PD detection in GIS.

## 1. Introduction

Partial discharge (PD) in gas-insulated switchgear (GIS) provides information for identifying insulation defects [1]. Optical emission offers an additional means of observing discharge activity alongside electromagnetic and acoustic detection [2,3]. Multispectral photodetector arrays have also been used to examine discharge characteristics in gas-insulated systems [4,5]. The optical detection chain relies on light collection, transmission, and photoelectric conversion. Losses before weak optical signals reach the photodetector limit the signal available for detection. Collecting light inside the equipment and delivering it to the receiver is therefore a primary consideration in optical sensor design.

Fluorescent optical fibers combine light sensing and transmission and have been used for PD detection [6,7,8]. Studies have examined fluorescent fiber sensing in SF_6_-insulated ring main units [9] and crystal-core fluorescent fibers for optical PD detection [10]. Coincidence single-photon counting has been used to suppress detector-noise false positives in cable-accessory measurements [11]. Using fluorescent fibers in online monitoring systems, however, requires secure mounting and sealing at the enclosure penetration.

A rigid lightguide rod coupled to a transmission fiber can extract light from the equipment and connect it to the external detection chain [12]. Lightguide rods have also been used to obtain phase-resolved patterns for defect diagnosis [13]. Optical–UHF sensing and fusion studies [14,15] also depend on effective delivery of the optical signal. For a lightguide rod installed in a GIS cover plate, the mounting bore, clamping length, and packaging boundary conditions jointly affect transmission. The output after installation cannot be assessed from material transmittance or front-section light collection alone.

Previous studies have reported favorable reception with a concave conical end face and with the front end face positioned flush with the inner GIS wall [12,16]. Light guides designed for direct reception by a photodetector can improve the irradiance distribution and field of view through front-end face geometry [17], whereas fluorescent light guides use wavelength conversion to improve spectral matching [18]. For a lightguide rod connected to an optical fiber, however, an increase in the radiant flux over the entire rear end face does not necessarily increase the flux received at the fiber entrance. Propagation losses in the metal-clamped rear section and the aperture mismatch at the exit impose two distinct constraints: shortening the rear section reduces losses, whereas tapering changes the spatial and angular distributions of the exiting light. Their effects therefore need to be evaluated under the actual packaging and finite-aperture reception conditions.

This study uses ray tracing to compare front-section parameters, rear-section length, front-end face shapes, and tapered rear-section profiles and to examine the effects of source position and contact boundaries. The rear-section designs are then evaluated through integrating sphere output measurements of four polymethyl methacrylate (PMMA) samples. Synchronized optical, ultrahigh-frequency (UHF), and ultrasonic measurements are subsequently used to examine the response of the optimized sample to needle–plate corona in a laboratory GIS model. These tests assess, respectively, the effect of the structure on output and its applicability to optical PD detection. The results provide a basis for assessing rear-section designs under finite-aperture reception conditions.

## 2. Lightguide Rod Structure and Optical Transmission

**Figure 1:**
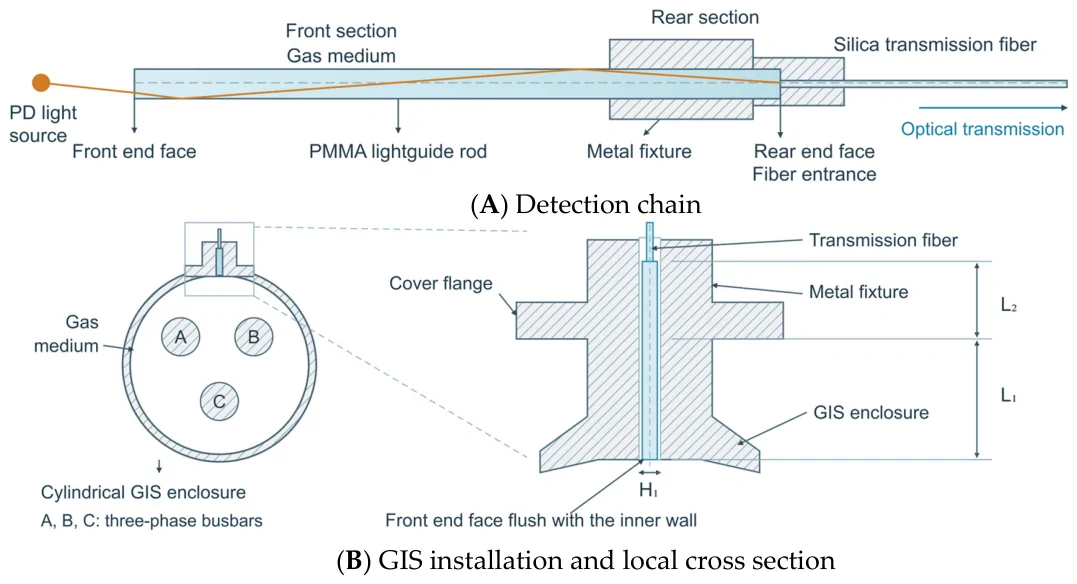
Optical detection chain using a lightguide rod and its installation in gas-insulated switchgear (GIS).

During PD, electrons accelerated by the local electric field can excite gas molecules or atoms through collisions. Radiative transitions of these excited species to lower energy states produce optical emission. The optical emission spectrum of PD depends on the gas medium, insulating materials, and discharge conditions [5,19,20]. The lightguide rod collects discharge light and guides it to the entrance of the transmission fiber. The fiber then carries the collected light to a photodetector, where it is converted into an electrical signal. The detection chain and its installation in the GIS are shown in Figure 1A,B, respectively.

The baseline lightguide rod is a cylinder with a diameter of 8 mm and a total length of 100 mm. It is installed in the GIS cover plate and connected to a transmission fiber, with its front-end face flush with the inner GIS wall [Figure 1B]. The rod is divided into front and rear sections at the start of the clamped region. The front section is unclamped, with its sidewall exposed to the surrounding gas medium, whereas the rear section forms the mounting region, with its sidewall in contact with the metal fixture. H_1_ denotes the mounting-bore diameter, and L_1_ and L_2_ denote the axial lengths of the front and rear sections, respectively. The front end face faces the GIS interior and serves as the light input, while the rear end face serves as the light output toward the transmission fiber. The model includes the lightguide rod, transmission fiber, mounting bore, and metal fixture, while omitting fasteners and other structural details that do not directly affect optical propagation.

The front-section sidewall is in contact with the gas medium. The refractive indices of the lightguide rod and gas are taken as *n*_1_ = 1.5 and *n*_2_ ≈ 1, respectively. For light incident from the rod onto the rod–gas interface, the incidence angle *θ*_1_ and refraction angle *θ*_2_ satisfy Snell’s law:

(1) *n*_1_ sin *θ*_1_ = *n*_2_ sin *θ*_2_.

Because *n*_1_ > *n*_2_, the critical angle for total internal reflection at this interface is

(2) $$\theta_{c}=\arcsin(\frac{n_{2}}{n_{1}})\approx 41.81{}^{\circ}.$$

At *θ*_1_ = *θ*_c_, the refraction angle is 90°. For *θ*_1_ > *θ*_c_, total internal reflection occurs at an ideally smooth, nonabsorbing interface [12].

The rear section is held by an aluminum fixture. The PMMA–aluminum contact interface is represented by an equivalent optical boundary in the ray-tracing model. Reflections at this interface change the ray paths and exit distribution, while repeated interactions accumulate absorption losses.

### 2.1. Front Section

**Figure 2:**
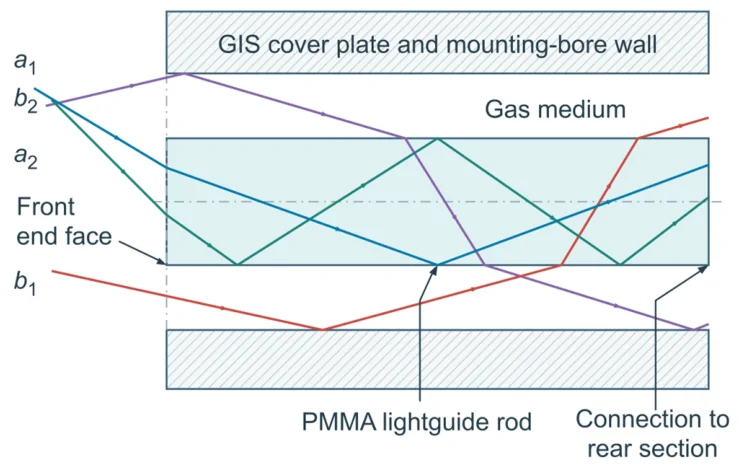
Representative ray paths in the front section of the lightguide rod.

Figure 2 illustrates representative ray paths for incidence through the front end face (a_1_ and a_2_) and through the bore clearance (b_1_ and b_2_). Whether directly incident rays propagate along the lightguide rod depends on the conditions for total internal reflection. Rays entering through the clearance can continue to propagate or escape after refraction at the sidewall. H_1_ affects obstruction and the collection range, whereas L_1_ affects the propagation paths through the installation channel and the number of reflections from the enclosure.

The simulations use H_1_ = 8.2, 9, 10, 12, 16, and 24 mm and L_1_ = 60, 50, 40, and 30 mm.

### 2.2. Rear Section

The rear section operates under different boundary conditions from the front section. It is tightly clamped by an aluminum fixture, and its sidewall is modeled with an equivalent optical boundary representing direct metal contact. When a ray reaches this sidewall, part of its energy is absorbed by the metal, while the remainder returns to the rod through reflection. With all other conditions held constant, increasing the rear-section length L_2_ increases the number of sidewall interactions and the accumulated absorption loss. Shortening L_2_ therefore helps reduce the optical transmission loss introduced by rear-section packaging. The lengths L_2_ = 10, 20, 30, and 40 mm are compared.

### 2.3. Front-End Face Shape

**Figure 3:**
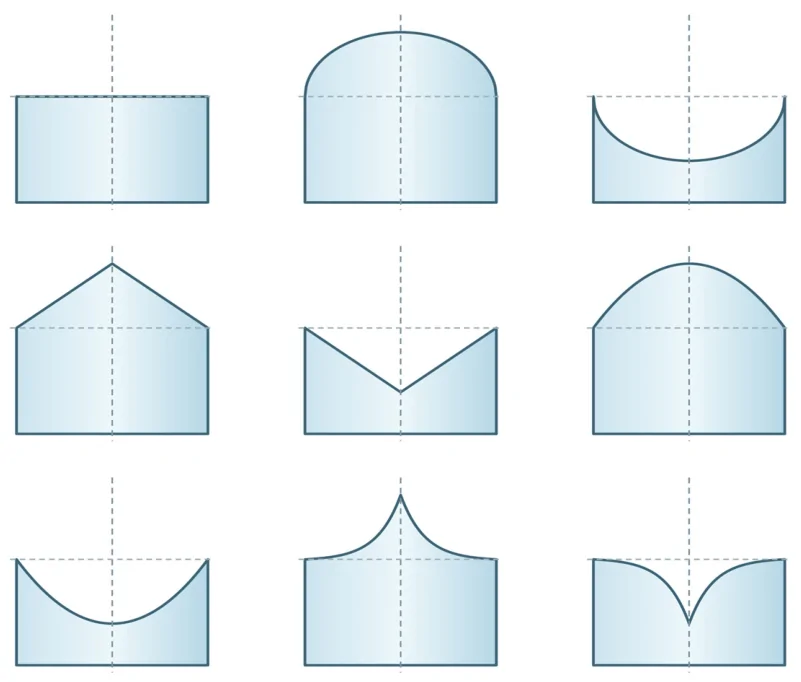
Nine axisymmetric front-end face shapes.

The front-end face shape affects optical propagation by changing the direction of refraction. A flat face is used as the baseline. Convex and concave spherical, conical, paraboloidal, and exponential surfaces are considered, giving nine axisymmetric shapes in total (Figure 3). Convex and concave refer to outward protrusion and inward recession, respectively. In the front-face comparisons, H is the axial height or depth of the vertex relative to the reference front plane, and α is the generatrix angle relative to that plane. This H is distinct from the rear-taper length defined in Section 2.4.

### 2.4. Tapered Rear-Section Profiles

**Figure 4:**
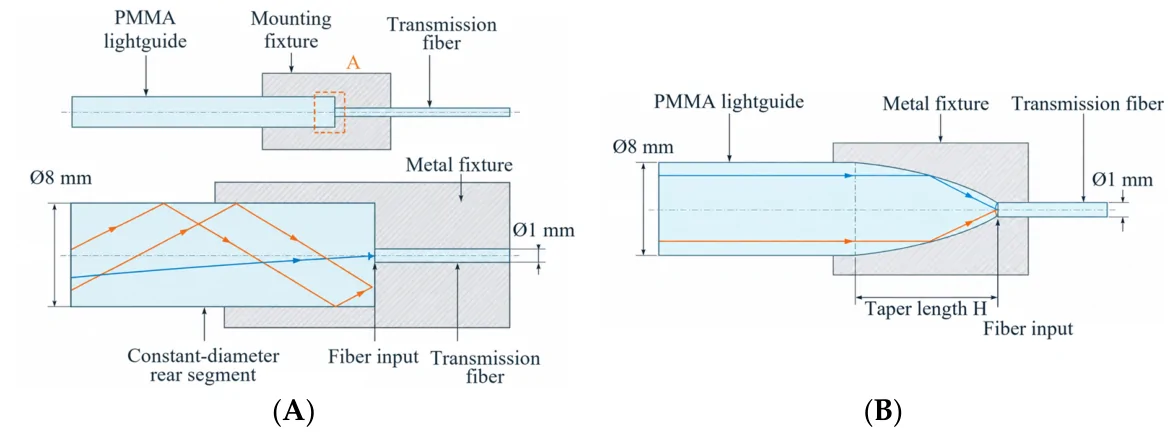
Coupling of constant-diameter and tapered rear sections to the fiber entrance: (**A**) Axial sectional view of the constant-diameter rear section. (**B**) Axial sectional view of the tapered rear section.

The 8 mm diameter lightguide rod and the 1 mm diameter fiber entrance have mismatched apertures. Only light exiting within the area corresponding to the fiber entrance is spatially positioned to enter the fiber directly [Figure 4A]. Tapering the rear section from 8 to 1 mm [Figure 4] changes the ray paths and the spatial and angular distributions of the exiting light, allowing some light that would otherwise miss the entrance to reach the receiving area.

**Figure 5:**
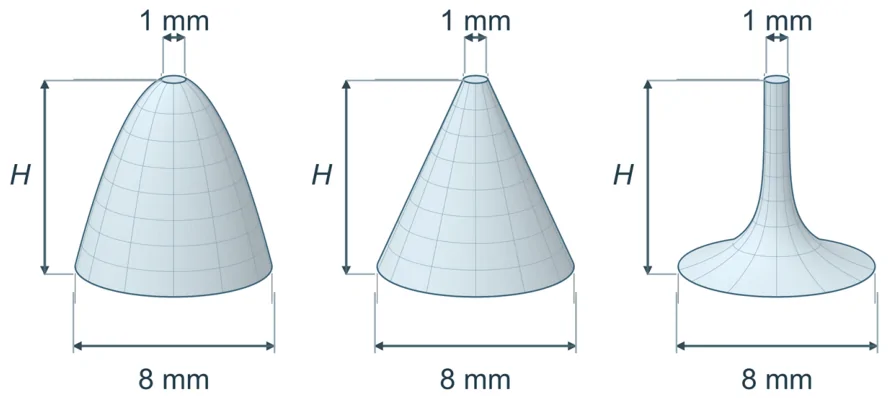
Three axisymmetric tapered rear-section profiles: parabolic, linear, and exponential, from left to right.

Figure 5 shows the three axisymmetric tapered profiles. The start of the taper is defined as z = 0, with the axial coordinate directed toward the exit. The cross-sectional radius is r(z), the taper length is H, and 0 ≤ z ≤ H. The initial and exit radii are r_0_ = 4 mm and r_1_ = 0.5 mm, respectively. The linear, parabolic, and exponential profiles are defined as

(3) $$r_{L}(z)=r_{0}-(r_{0}-r_{1})\frac{z}{H},$$

(4) $$r_{P}(z)=\sqrt{{r_{0}}^{2}-({r_{0}}^{2}-{r_{1}}^{2})\frac{z}{H}},$$

(5) $$r_{E}(z)=r_{1}+(r_{0}-r_{1})\frac{e^{-\frac{z}{l}}-e^{-\frac{H}{l}}}{1-e^{-\frac{H}{l}}}.$$

The subscripts L, P, and E denote the linear, parabolic, and exponential profiles, respectively, and ℓ = 1 mm is the characteristic length of the exponential profile. All three profiles satisfy r(0) = r_0_ and r(H) = r_1_. For the parabolic profile, r^2^ varies linearly with z. The radius is continuous at the junction between the taper and the constant-diameter section, but the tangent is discontinuous.

The profile comparison maintains a total length of 100 mm, L_1_ = 60 mm, and L_2_ = 40 mm, with H = 4, 8, 12, 16, and 20 mm. The constant-diameter portion of the rear section has a length of L_2_ − H, and the axial position of the exit remains fixed.

**Figure 6:**
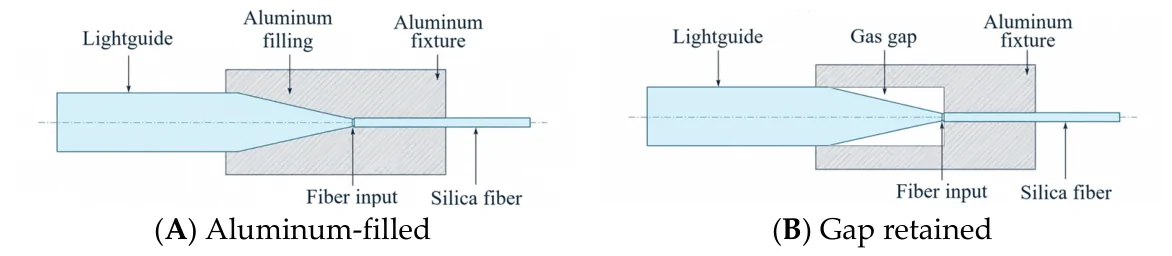
Two packaging configurations for the tapered rear section.

The gap between the tapered sidewall and the constant-diameter fixture is either filled with aluminum or retained as a gas gap (Figure 6). With aluminum fill, the aluminum alloy contacts the tapered sidewall. With the gas gap retained, no metal is added to the gap. The profiles and dimensions are identical, so the comparison isolates the effect of the local contact boundary on the radiant flux received at the fiber entrance.

## 3. Simulation Analysis

### 3.1. Simulation Model

Ray tracing was performed in TracePro Expert 7.3.4. The PD emission region is approximated by a spherical source with a diameter of 0.1 mm that emits light uniformly outward along its surface normals. Five wavelengths of 300, 350, 400, 450, and 500 nm are assigned equal power weights. The total radiant power is 5 W, and 2,000,000 rays are traced.

**Figure 7:**
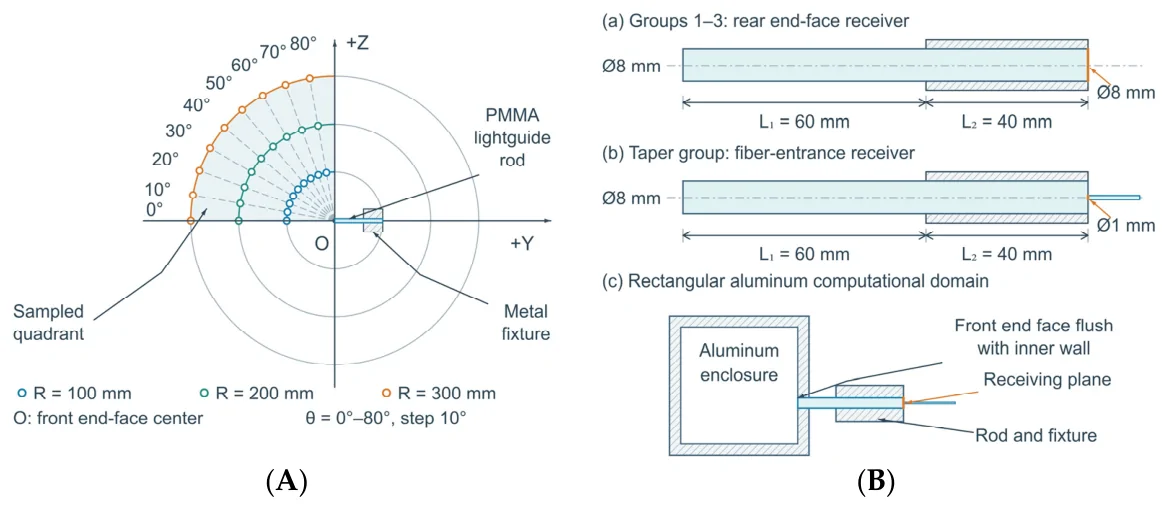
Source positions, receiving planes, and computational space in the simulations: (**A**) Source positions and geometry. (**B**) Receiving planes and computational domain

The center of the front end face is defined as the origin, with the positive Y-axis along the rod axis. The source lies in the half-space inside the GIS. Its angular position in the Y–Z plane is specified by θ, the angle between the source direction and the negative Y-axis. The first three structural comparison groups each use 9 source positions at a fixed source distance of 100 mm, whereas the tapered rear-section profile group uses 27 source positions. In both cases, θ ranges from 0° to 80° in 10° increments. For the tapered rear-section profile group, the nine angles are evaluated at source distances of 100, 200, and 300 mm [Figure 7A].

The mounting position of the rod is held fixed while the source position is varied to evaluate the effects of relative distance and direction on optical reception. The same source positions are used for all geometries within each comparison group, providing common illumination conditions for structural assessment.

The source is used solely to provide consistent conditions for structural comparisons. The simulated received quantities do not directly represent the actual discharge intensity or detection sensitivity.

The optical transmission of PMMA depends on wavelength, as documented by published spectral absorption measurements [21]. Material properties affect the attenuation and refraction of individual spectral components, while geometry affects propagation paths, interface incidence angles, reflection counts, and reception at the fiber entrance. This study holds PMMA and the reference spectrum fixed across geometries and varies rear-section length, taper profile, and contact boundaries. The five equal-power wavelength components provide a common input for structural comparison, without fitting the source to a specific PD emission spectrum.

The lightguide rod is made of PMMA. The default material and surface optical properties in TracePro were used for PMMA and the aluminum-contact boundary and were kept unchanged across the structural comparisons. The baseline structure has L_1_ = 60 mm and L_2_ = 40 mm, with planar front and rear end faces; the remaining baseline dimensions are given in Section 2. Figure 7B illustrates the two receiving planes and their spatial relationship to the enclosure.

The first three simulation groups evaluate the radiant flux received over the entire rear end face, which has a diameter of 8 mm. The tapered rear-section profile group evaluates the radiant flux received at a 1 mm diameter fiber entrance placed directly against the rear end face. These quantities describe flux at the receiving planes and do not establish the power transmitted through the fiber. The receiving planes and computational space are shown in Figure 7B.

### 3.2. Simulation Results

The baseline structure has a diameter of 8 mm, H_1_ = 8.2 mm, L_1_ = 60 mm, and L_2_ = 40 mm, with flat front and rear end faces. The rear-end-face and fiber-entrance results are normalized separately by the corresponding mean received radiant flux of this baseline over the source positions used for each comparison.

#### 3.2.1. Front-Section Dimensions

Table 1 lists the received radiant flux statistics for each combination of front-section dimensions, all normalized by the common rear-end-face baseline defined in Section 3.2. Increasing H_1_ from 8.2 to 24 mm raises the mean by 2.56%, 2.26%, 2.21%, and 1.44% for L_1_ = 30, 40, 50, and 60 mm, respectively. At a given H_1_, the maximum relative difference between the means for different L_1_ values is no greater than 1.33%, and the ranges of received flux are similar. Over the same H_1_ range, the maxima in the four L_1_ groups increase by 1.74–2.32%. The L_1_ = 40 mm group consistently exhibits the highest mean, whereas the L_1_ = 60 mm group has the highest maximum. This difference in ranking indicates that the geometry yielding the highest peak reception does not necessarily provide the highest average reception across the sampled source positions. Overall, within the investigated dimensional ranges and simulation conditions, adjusting the front-section dimensions has only a small effect on the mean received radiant flux.

#### 3.2.2. Rear-Section Length

**Figure 8:**
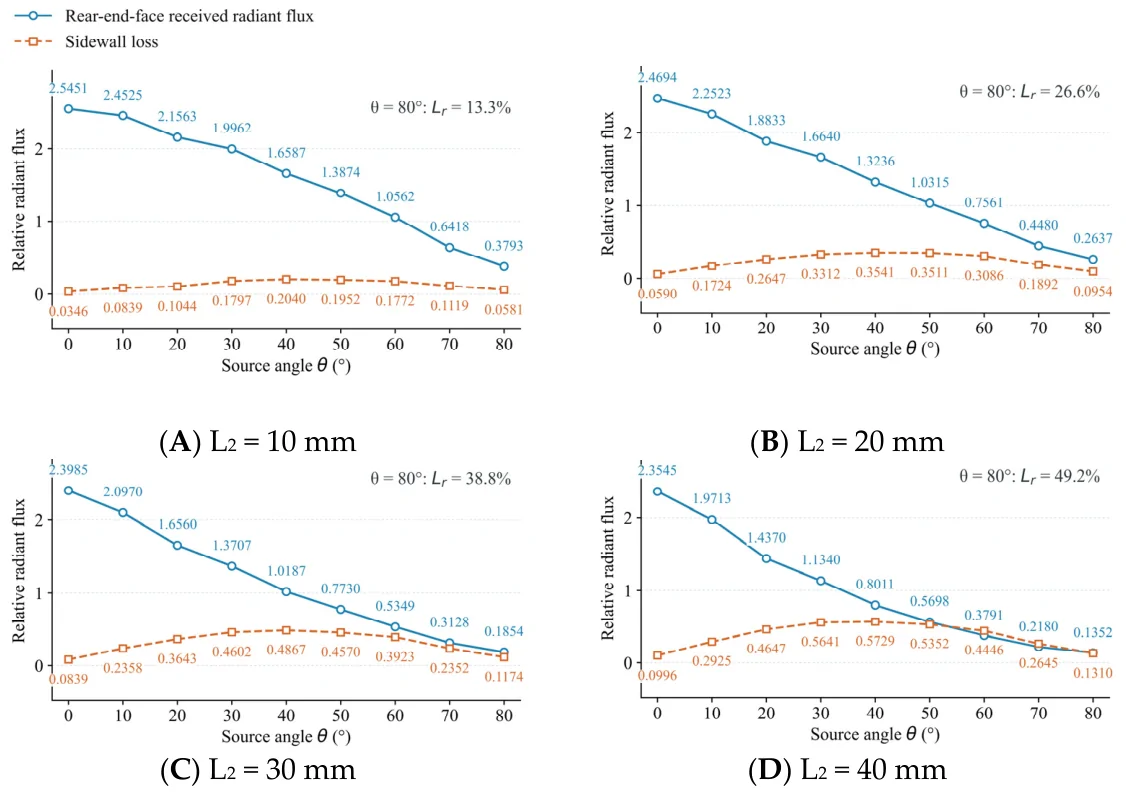
Effects of rear-section length on the radiant flux received at the rear end face and sidewall loss.

In Figure 8, the received radiant flux decreases with increasing source angle for all four rear-section lengths. At a given source position, increasing L_2_ reduces the received flux and increases the sidewall loss; both quantities are normalized by the same baseline. This trend is consistent with greater cumulative absorption at the metal-contact sidewall in a longer rear section. Reducing L_2_ from 40 to 10 mm increases the mean received flux over the 9 source positions by 58.6%. The increases at θ = 0° and 80° are 8.1% and 180.5%, respectively, indicating a greater relative improvement at the larger source angle.

The minimum received radiant flux occurs at θ = 80° for all rear-section lengths. The rear-section sidewall loss fraction Lᵣ at this position is defined as

(6) $$L_{r}=\frac{\Phi_{\mathrm{loss}}}{\Phi_{\det}+\Phi_{\mathrm{loss}}}\times 100\%,$$

where Φdet and Φloss are the normalized radiant flux received at the rear end face and lost at the rear-section sidewall, respectively. Lᵣ is the fraction of sidewall loss in the sum of these two quantities and does not represent the total transmission loss of the lightguide rod. The loss fractions at θ = 80° are annotated in the corresponding panels of Figure 8.

As L_2_ increases from 10 to 40 mm, Lᵣ at θ = 80° rises from 13.3% to 49.2%, consistent with the accumulation of sidewall losses in a longer rear section in contact with metal. More frequent ray–sidewall interactions affect reception at the rear end face through interfacial absorption and changes in reflection direction. For the specified packaging and the investigated range of 10–40 mm, the rear-section length should therefore be minimized while satisfying clamping, mechanical strength, and sealing requirements.

#### 3.2.3. Front-End Face Shape

**Figure 9:**
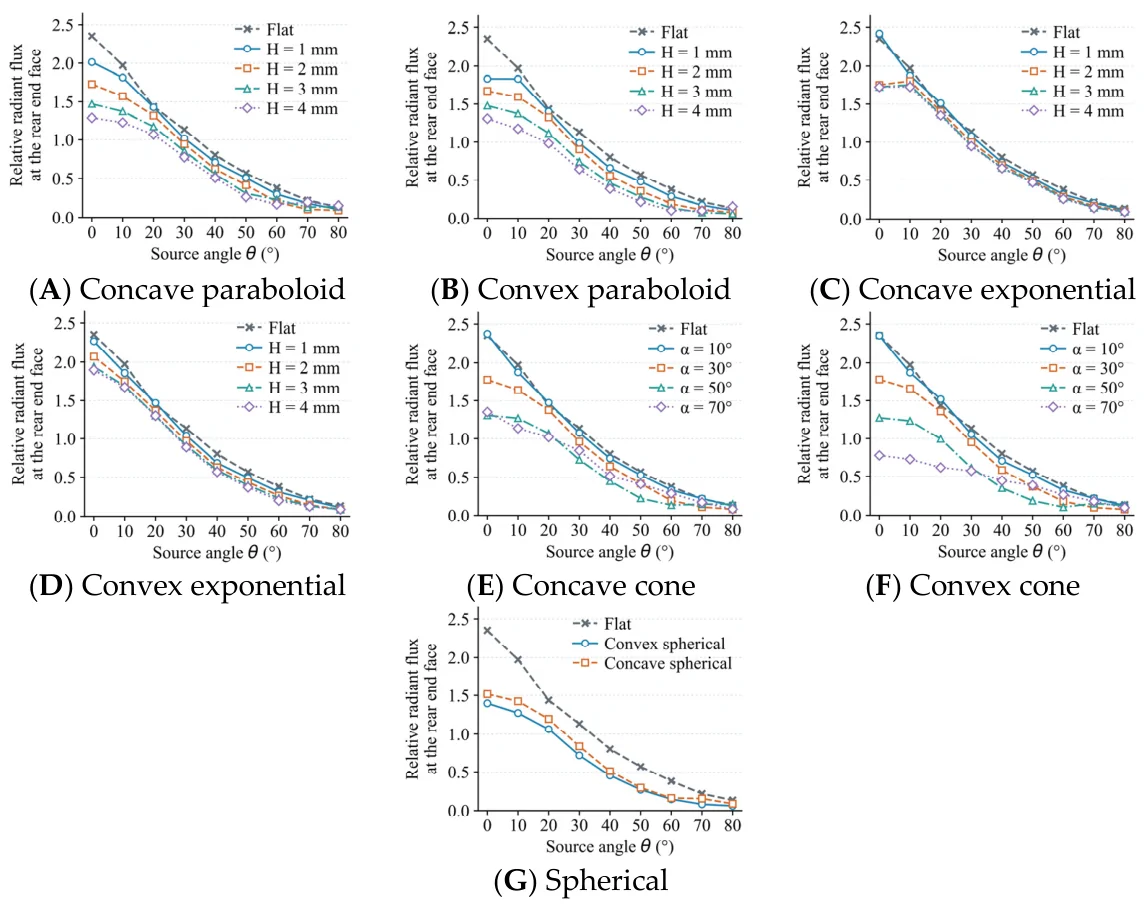
Effect of front-end face shape on the normalized radiant flux received at the rear end face.

Figure 9 shows that the more complex front end faces examined do not provide a consistent overall reception advantage. Changing the surface geometry modifies the refraction direction of incident rays and their subsequent propagation paths but does not necessarily increase the flux reaching the rear end face. Conical surfaces with small generatrix angles α produce responses close to that of the flat face. The maximum received flux of the 10° concave conical surface is approximately 0.8% higher than that of the flat face, but its mean over the 9 source positions is approximately 2.8% lower. Thus, the modest peak gain does not translate into improved average reception across the sampled positions. The larger generatrix angles examined yielded lower mean received flux than α = 10°; the maximum for the 70° convex conical surface falls to 33.0% of that of the flat face. For the exponential surfaces, the mean received flux decreases over the H values examined, where H denotes the axial height or depth of the surface vertex relative to the reference front plane. No overall reception advantage is obtained. All the paraboloidal surfaces examined yield lower mean received flux than the flat face. Considering the reception results within the investigated shape and size ranges, together with ease of fabrication, a flat front end face is recommended.

#### 3.2.4. Tapered Rear-Section Profiles

**Figure 10:**
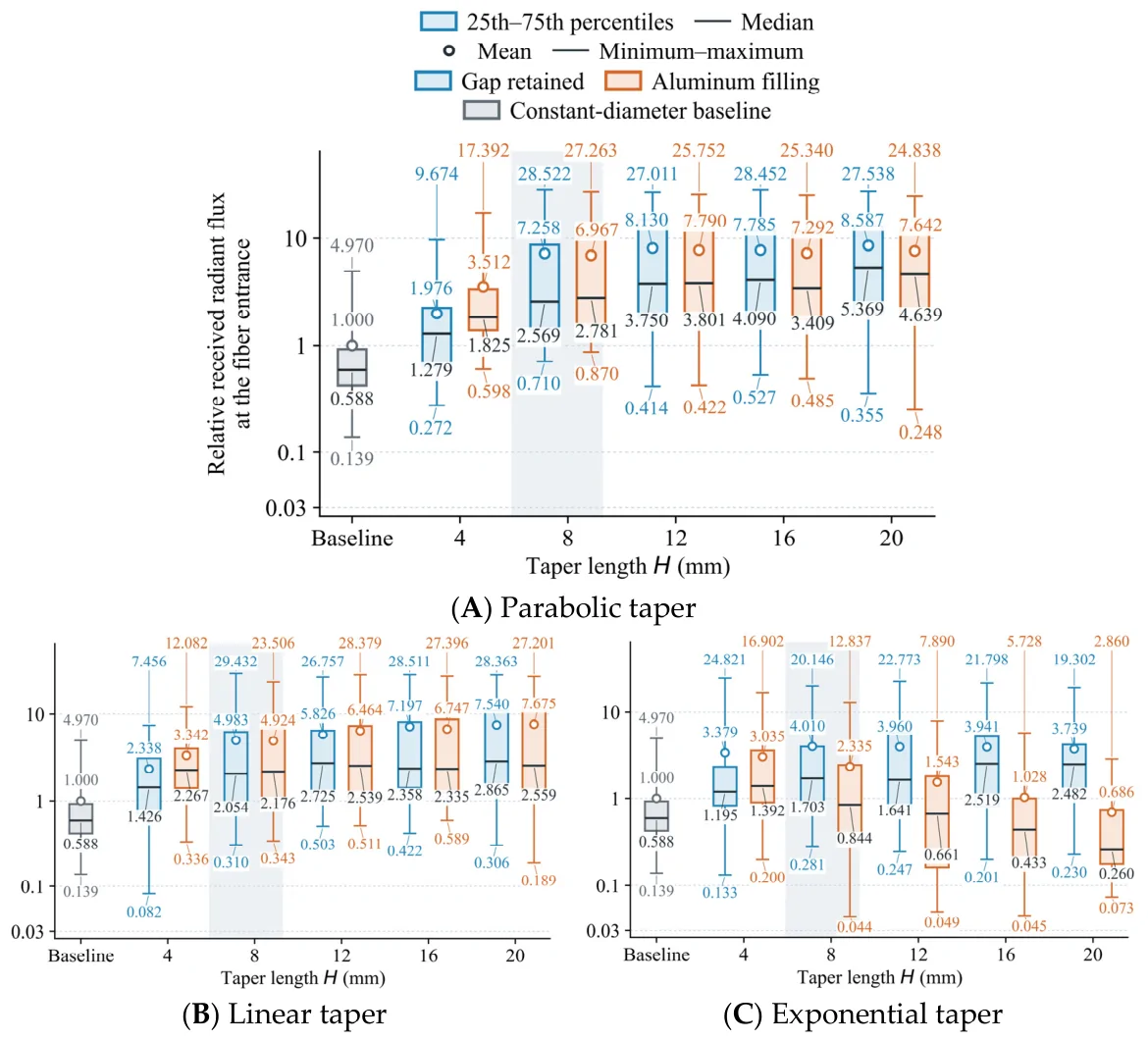
Effect of the rear taper profile on the relative radiant flux received at the fiber entrance.

Figure 10 compares the radiant flux received at the fiber entrance for the three taper profiles with either a retained gas gap or aluminum filling. The maximum, mean, median, and minimum values are explicitly labeled for each design. The mean values for the parabolic and linear tapers generally increased with H, although the parabolic profile exhibited fluctuations at some lengths. For the exponential taper with a retained gas gap, the mean reached 4.010 at H = 8 mm and then decreased slightly. With aluminum filling, the mean for the exponential taper decreased continuously; at H = 20 mm, its maximum, mean, and median were all below those of the constant-diameter baseline. At H = 4 mm, aluminum filling increased the mean values for both the parabolic and linear tapers. For the parabolic taper at H = 8 mm, however, it reduced the mean by approximately 4.0% while increasing the minimum from 0.710 to 0.870. The effects of packaging on the mean and minimum received flux therefore differed and depended on both the profile and H.

Under each packaging condition, the parabolic taper with H = 8 mm yielded the largest minimum received flux among the three profiles and five taper lengths examined under the reference spectrum and source positions defined in Section 3.1 (Figure 10). With the gas gap retained, its mean and minimum values were 7.258 and 0.710, respectively. Increasing H to 20 mm raised the mean to 8.587 but reduced the minimum to 0.355, indicating a trade-off between average reception and reception at the least favorable sampled position. Considering both the minimum received flux and fixture manufacturing requirements, the parabolic taper with H = 8 mm and a retained gas gap was selected for sample testing.

For the selected parabolic taper with H = 8 mm and a retained gas gap, the mean radiant flux received at the fiber entrance was 5.65, 11.41, and 9.74 times that of the constant-diameter baseline at source distances of 100, 200, and 300 mm, respectively. At each distance, the mean was calculated over the nine angular positions for each geometry. These results show improved angularly averaged reception at all three investigated distances.

## 4. Experimental Validation

### 4.1. Integrating Sphere Comparison of PMMA Samples

#### 4.1.1. Samples and Test Procedure

**Figure 11:**
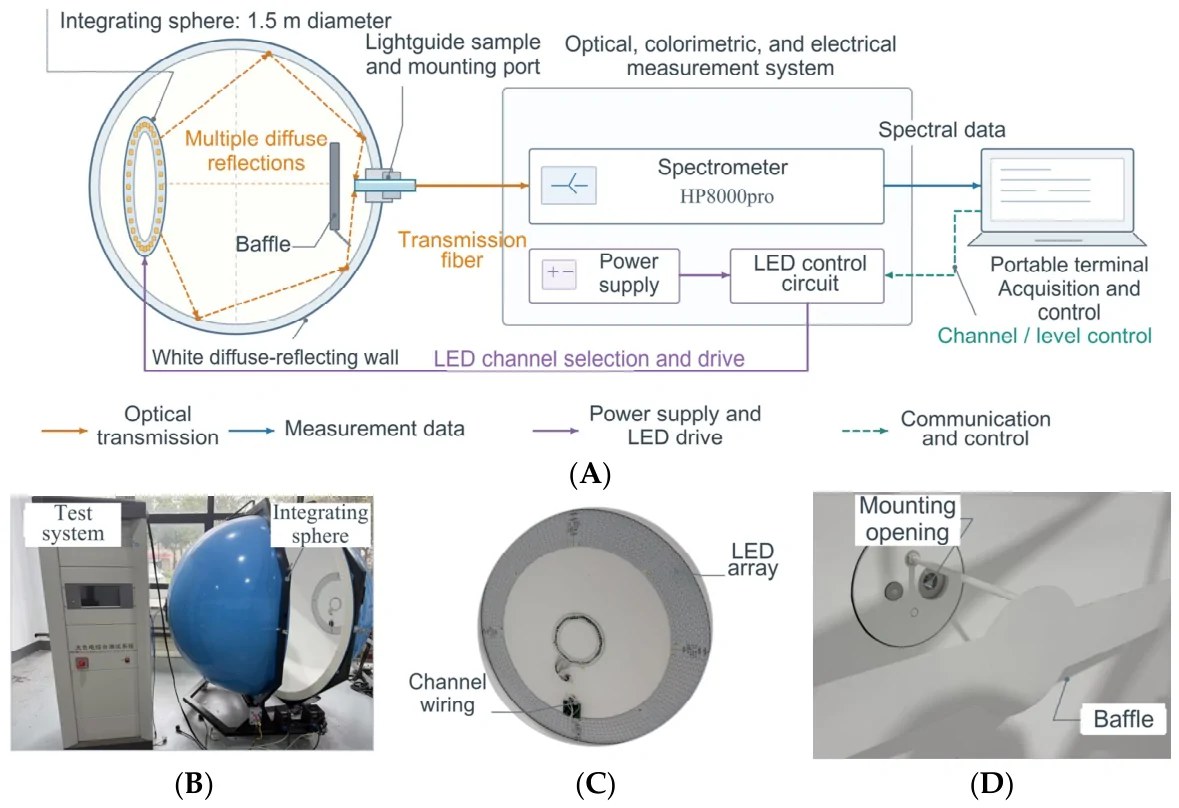
Integrating sphere test setup and key components. LED denotes a light-emitting diode: (**A**) Experimental setup and measurement chain. (**B**) Setup and internal arrangement. (**C**) Annular LED board. (**D**) Mounting port and baffle.

Integrating sphere tests compared the detector output of four PMMA samples under common illumination and fiber reception conditions. The setup consisted of an integrating sphere 1.5 m in diameter with a diffusely reflecting inner surface, an annular light-emitting diode (LED) board, a sample mounting assembly, a transmission fiber, and an integrated optical, colorimetric, and electrical measurement system (Figure 11). A customized HP8000pro spectrometer measured the output spectra, and the setup included a 310 nm ultraviolet channel. Multiple diffuse reflections from the sphere’s wall mixed the internal light field. A baffle in front of the port reduced direct illumination from the light source. The LED board, baffle, and mounting position were kept unchanged when the samples were exchanged.

**Figure 12:**
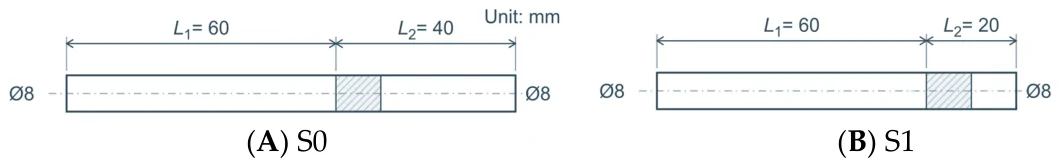

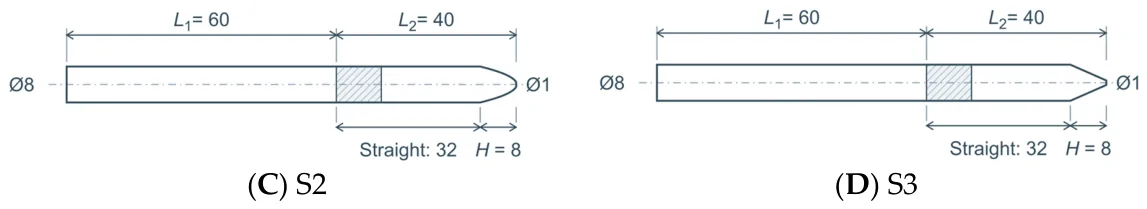
Nominal dimensions of the four polymethyl methacrylate (PMMA) lightguide rod samples. Hatched regions indicate the common clamping region.

One PMMA sample was tested for each geometry: S0 (long constant-diameter rear section), S1 (short constant-diameter rear section), S2 (parabolic taper), and S3 (linear taper). The samples were manufactured by a contractor according to the nominal dimensions. All samples had flat front and rear end faces. Their dimensions and common clamping region are shown in Table 2 and Figure 12. The S1/S0 comparison evaluates the effect of rear-section length, S2/S0 evaluates the combined effect of the taper design, and S2/S3 compares the taper profiles.

Eight LED channels with peak wavelengths of 310, 365, 405, 450, 505, 610, 660, and 730 nm were tested at five power-setting levels of 100, 300, 500, 700, and 900, giving 40 illumination conditions. The four samples were measured sequentially under each condition. The integrating sphere was closed, and the same mounting and shielding conditions were restored for each measurement. A dark background recorded with the light source switched off was subtracted from each spectrum.

The detector output was characterized by automatic spectral integration in the spectrometer software after dark-background subtraction. For each LED channel, a common integration interval covering the emission band was used for all four samples. Let *B* denote the set of wavelength sample indices within this interval. The integrated response of sample *i* is

(7) $$Q_{i}=\sum_{k\in B}[C_{i,k}-C_{d,k}]{\Delta \lambda }_{k},$$

where *C_i_*_,_*_k_* is the spectral reading of sample *i* before dark-background subtraction, and *C*_d,_*_k_* is the dark-background reading at wavelength sample *k*; *Δλ_k_* is the wavelength interval. The response at LED peak wavelength *λ* and power-setting level *u* is denoted by *Q_i_*(*λ*, *u*). Taking the response of S0 at 310 nm and level 100 as the common reference *Q*_ref_, the normalized response is defined as

(8) $$R_{i}(\lambda ,\;u)=\frac{Q_{i}(\lambda ,\;u)}{Q_{\mathrm{ref}}}.$$

The normalized response of S0 under the reference condition is unity. All 160 responses use this common reference, preserving the relative amplitudes across geometries, LED channels, and power-setting levels. The output ratio between two samples under the same illumination condition is defined as

(9) $$G_{i/j}(\lambda ,\;u)=\frac{R_{i}(\lambda ,\;u)}{R_{j}(\lambda ,\;u)}=\frac{Q_{i}(\lambda ,\;u)}{Q_{j}(\lambda ,\;u)},$$

where *i* and *j* identify the samples being compared, and *G_i_*_/_*_j_* > 1 indicates that sample *i* has a higher output than sample *j*. Each ratio is calculated at the same LED channel and power-setting level and is unaffected by the common normalization. The ratios are summarized using their equally weighted arithmetic mean over the 40 illumination conditions.

#### 4.1.2. Detector Output and Structural Comparison

**Figure 13:**
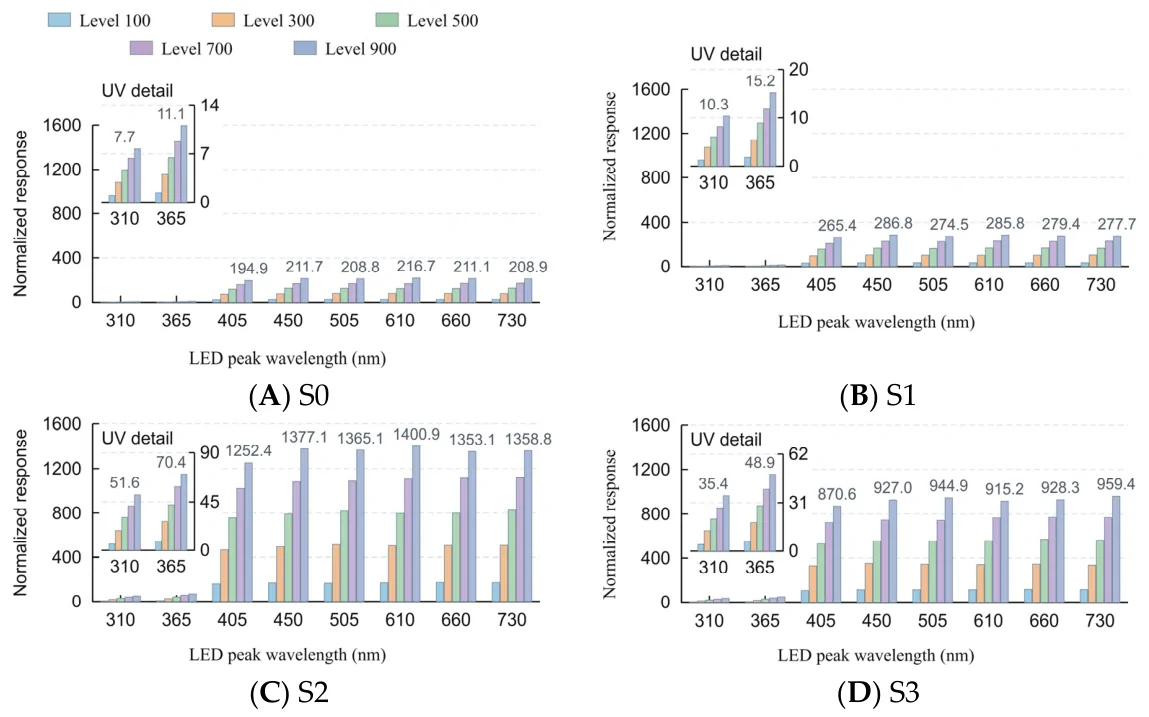
Normalized responses of the four PMMA lightguide rod samples under all illumination conditions. The four panels share the same response scale; insets show enlarged views of the 310 and 365 nm channels.

Figure 13 shows the normalized responses of all four samples under all illumination conditions. At every tested wavelength, the response increased with the power-setting level, and the output ranking S2 > S3 > S1 > S0 was maintained across all 40 conditions. The maximum response of each sample at each wavelength occurred at setting 900; these values are labeled in the figure. Insets provide enlarged views of the 310 and 365 nm channels.

**Figure 14:**
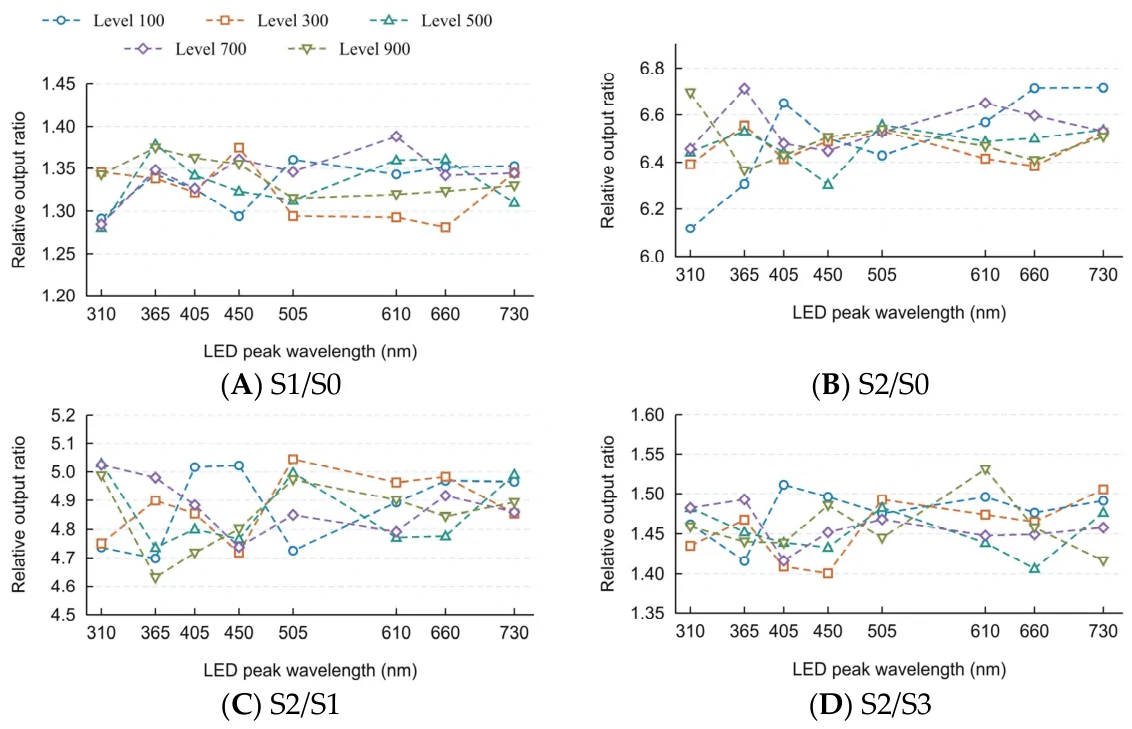
Relative output ratios between samples at the same LED channel and power-setting level.

Figure 14 shows the output ratios under matching illumination conditions. Each ratio was calculated from outputs measured at the same LED channel and power-setting level. The main geometric difference between S1 and S0 is the reduction in constant-diameter rear-section length from 40 to 20 mm. The S1/S0 ratio ranged from 1.28 to 1.39, with an equally weighted mean of 1.33 across the 40 conditions, corresponding to an average relative output increase of approximately 33%. This follows the same direction as the simulated rear-length trend.

For samples of equal total length, the S2/S0 and S3/S0 ratios ranged from 6.12 to 6.72 and from 4.18 to 4.63, respectively, with equally weighted means of 6.50 and 4.45. These comparisons involve simultaneous changes in taper profile and exit diameter and therefore reflect the combined effect of the taper design. Both tapered samples showed larger output gains relative to S0 than those obtained by shortening the constant-diameter rear section under the tested conditions. Despite its longer rear section, S2 also produced an output 4.63–5.05 times that of S1. However, the two samples differ in rear-section length, shape, and exit diameter, so this ratio reflects the joint effect of several geometric changes.

S2 and S3 have the same total length, rear-section length, taper length, and entrance and exit diameters; their main geometric difference is the taper profile. With these dimensions matched, the S2/S3 comparison provides a more direct assessment of taper-profile effects. The S2/S3 ratio ranged from 1.40 to 1.53 across the 40 conditions, corresponding to a 40–53% increase in output. Its equally weighted mean of 1.46 represents an average relative output increase of approximately 46%. Thus, for H = 8 mm and the fiber reception conditions used here, the parabolic taper consistently produced a higher output than the linear taper.

For each LED channel, the mean output ratio was also calculated over its five power-setting levels. These channel means ranged from 6.42 to 6.56 for S2/S0 and from 1.44 to 1.48 for S2/S3. The unchanged sample ranking and the small variation in channel means show that the relative advantage of the selected parabolic sample persisted across the tested LED emission bands under common reception conditions.

Manufacturing deviations can change the rod dimensions and local surface profiles. With front-face planarity and orientation maintained, a small axial deviation mainly changes the entrance position and propagation distance. The rear-section designs differ in length or contraction profile. Small dimensional deviations relative to the simulated parameter intervals preserve the main distinctions between the length designs. Local profile errors may alter incidence angles and ray exit distributions while retaining the taper’s collection mechanism if the overall profile, exit aperture, and coupling alignment are preserved. The simulated metric integrates radiant flux over the receiving area, so changes to individual ray paths do not necessarily remove the overall collection benefit. The output advantage of the fabricated parabolic sample under all 40 illumination conditions shows that the structural benefit was retained after fabrication.

S2 was therefore selected for the GIS tests to evaluate its optical response under actual discharge conditions. Its dimensions are listed in Table 2.

### 4.2. Corona Discharge Responses in GIS

#### 4.2.1. Experimental Setup and Data Processing

**Figure 15:**
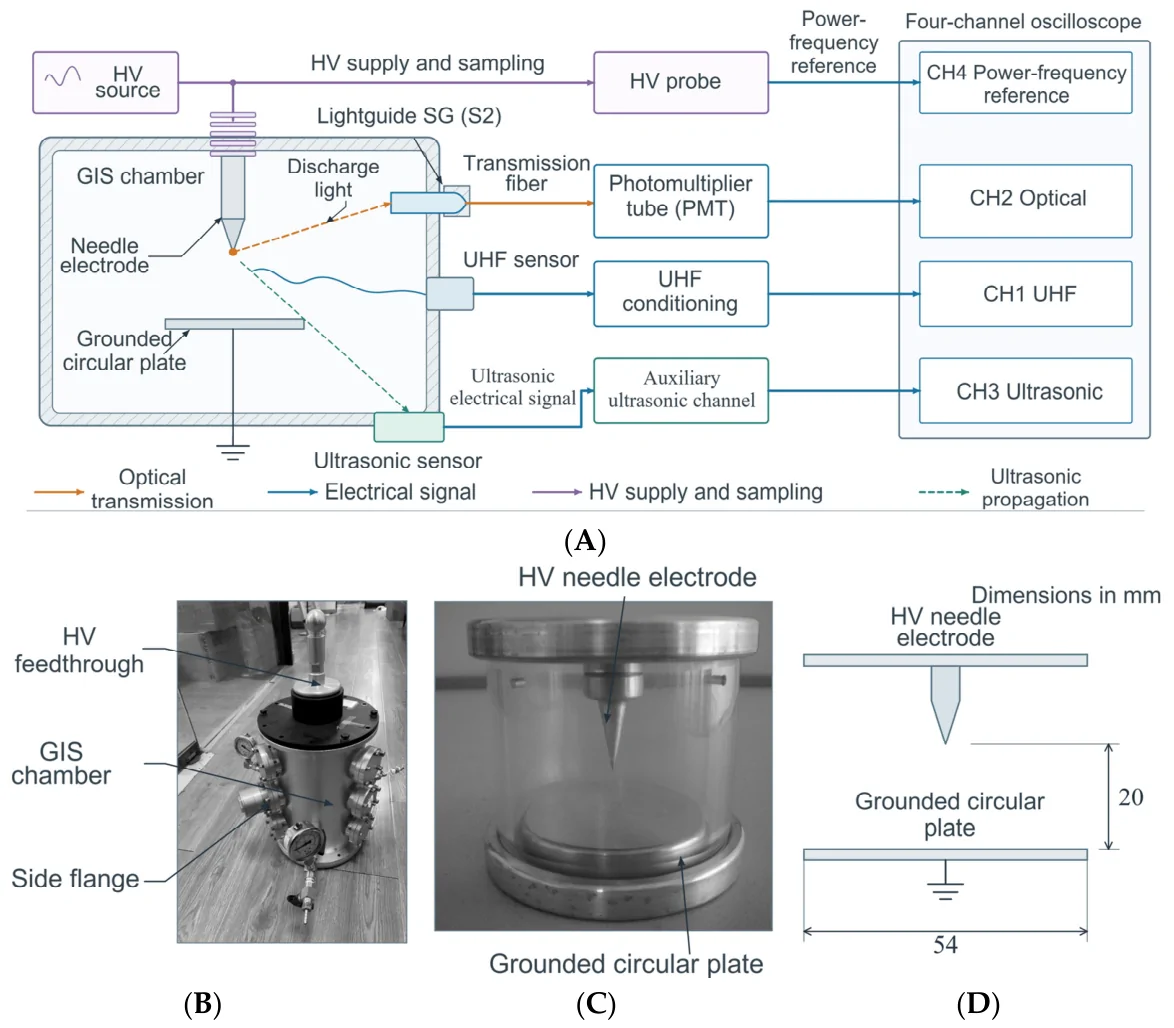
GIS needle–plate corona test setup, defect geometry, and synchronous acquisition chain. UHF denotes ultrahigh frequency: (**A**) GIS needle–plate corona setup and synchronized acquisition. (**B**) GIS experimental setup. (**C**) Needle–plate corona defect. (**D**) Needle–plate geometry.

S2 was used in needle–plate corona tests in air at 1 atm in a laboratory GIS model, using stepwise voltage increases and representative test levels of 8.8 and 12 kV (Figure 15, where SG denotes S2). The sample remained at a fixed mounting position throughout these tests. The sample was connected to a photomultiplier tube through a transmission fiber. An oscilloscope simultaneously recorded the UHF signal (CH1), optical signal (CH2), signal from the auxiliary ultrasonic channel (CH3), and power-frequency reference from a high-voltage probe (CH4). The synchronized records enabled temporal comparison of the optical and UHF responses, while CH4 provided the power-frequency phase reference for subsequent analysis. Ten consecutive records were analyzed at each voltage level. Each record contained 10,000 exported time bins per channel, with a 10 μs interval between time stamps and a recording window of approximately 100 ms.

Previous lightguide-rod studies have detected corona, surface, floating, and particle discharges in SF_6_ [12,13]. Needle–plate corona was selected here because its polarity-dependent activity and its evolution with voltage allow stronger and weaker responses to be compared within one defect configuration. The rod, defect, and their relative mounting positions were unchanged during stepwise voltage increases. The 8.8 kV records represent a weaker discharge stage, while 12 kV represents a stronger pre-breakdown stage. Further voltage increases led to breakdown under this needle–plate configuration in air. These voltages characterize the tested discharge stages and do not specify the maximum operating voltage of the lightguide rod.

The PMT control setting was adjusted to obtain an output amplitude suitable for oscilloscope acquisition and remained unchanged across the two voltage levels and within each set of ten consecutive records. The corresponding gain was not recorded. The optical measurement chain was not calibrated against a known optical input, and the optical-channel amplitudes are reported as detector output voltages.

For each record, the sequence of midpoint values between the CH4 minimum and maximum voltages in each time bin was fitted with a sinusoidal function including a dc offset:

(10) *v*_4_(*t*) = *c* + *A* sin(2*πft* + *ψ*),

where *c* is the dc offset, *A* is the power-frequency ac amplitude, *f* is the frequency, *ψ* is the initial phase, and *t* is time. The frequency *f* was estimated within 48–52 Hz. The positive-going zero crossing of the fitted ac component was taken as the phase origin. The phase of time bin *j* is *φ_j_* = (2*πft_j_* + *ψ*) mod 2*π*, where *t_j_* is its time stamp. The resulting phase values were converted to degrees for statistical analysis. This procedure does not resolve the exact phase of an individual pulse within a time bin. The 10 μs interval is the spacing of the exported extrema bins and was not specifically optimized for precise single-pulse phase assignment. At 50 Hz, it corresponds to a nominal phase span of 0.18°, which is not a validated single-pulse phase accuracy. The occurrence times of the extrema within each bin were not stored, so extrema in the same bin of different channels do not establish pulse-by-pulse coincidence.

For each record and each of the UHF and optical channels, the median of the midpoint sequence between the time-bin minimum and maximum voltages was used as the baseline *b*. The envelope amplitude is defined as

(11) *A_j_* = max(|*u_j_*_,min_ − *b*|, |*u_j_*_,max_ − *b*|),

where *u_j_*_,min_ and *u_j_*_,max_ are the minimum and maximum voltages in time bin *j*, respectively. The phase distributions use 5° phase bins. For 12 kV, the UHF and optical amplitude ranges are 0–1 V and 0–3 V, respectively. For 8.8 kV, the corresponding ranges are 0–0.2 V and 0–0.4 V. Each range is divided into 80 equal-width amplitude bins. For each channel at each voltage level, 100,000 time bins from the ten records, corresponding to approximately 1 s, were pooled separately. The color scale has 70 discrete levels and indicates time-bin count on a logarithmic scale; these counts do not represent independent discharge pulses.

#### 4.2.2. Discharge Responses and Phase Distributions

**Figure 16:**
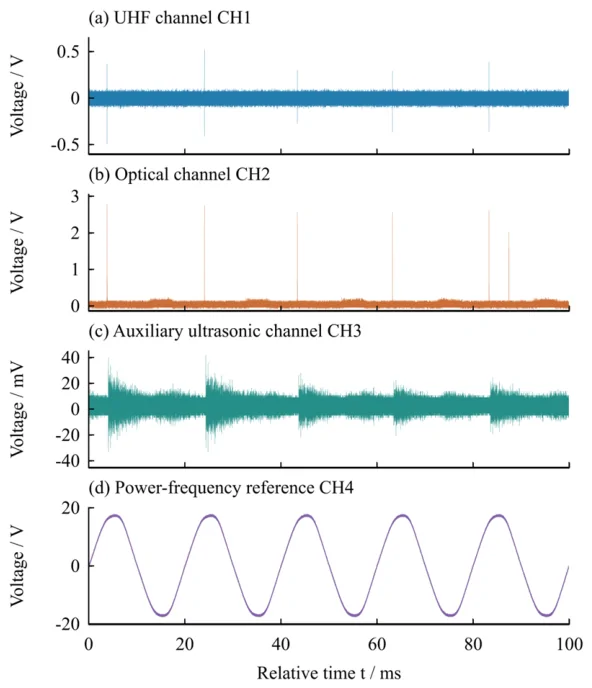
Multichannel envelopes of voltage extrema within time bins for the 12 kV needle–plate corona record a6.

In Figure 16, time zero is the start of the record. The major UHF envelope peaks recur at intervals of approximately one power-frequency period, with optical responses in similar time intervals. The optical channel also records repeated clusters of low-amplitude responses during the negative half-cycle, whereas comparable UHF responses are not clearly resolved in the corresponding intervals. Ultrasonic activity in nearby time intervals provides supporting evidence. The S2 optical detection chain therefore preserves the timing of the main activity and provides complementary information on weak activity that is difficult to distinguish in these UHF records.

**Figure 17:**
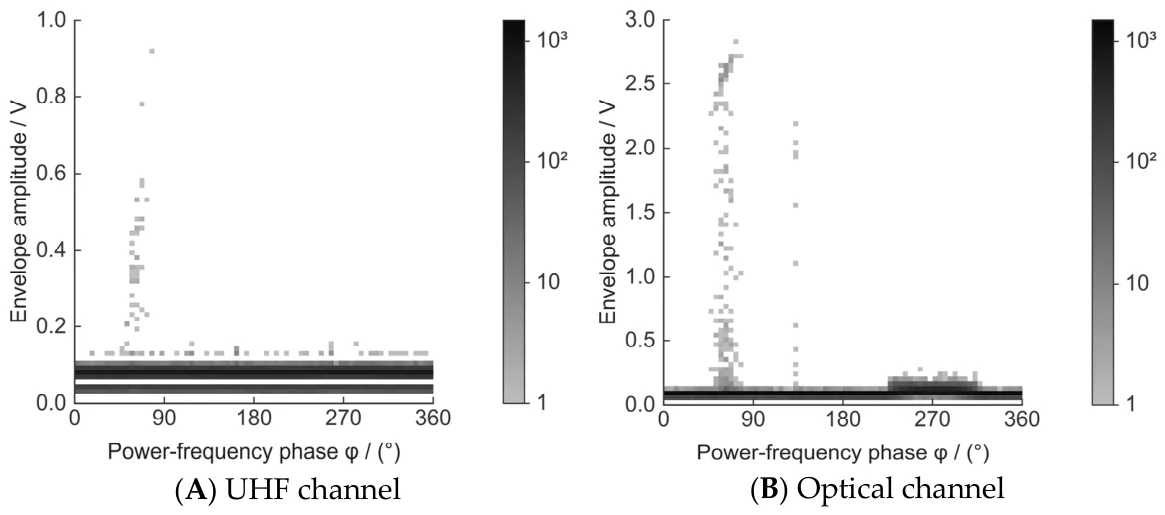
Envelope amplitude distributions referenced to the synchronously recorded power-frequency signal.

In Figure 17, larger UHF and optical envelope amplitudes are concentrated mainly at 45–90°, exhibiting similar power-frequency phase clustering consistent with the time-domain envelope analysis. The optical channel also shows a larger response in the 130–135° phase bin. Low-amplitude optical responses at approximately 225–320° during the negative half-cycle correspond to the clusters in Figure 16. The ultrasonic channel records acoustic vibration responses and provides auxiliary evidence of acoustic activity. The optical channel provides complementary observations of low-amplitude activity under the tested conditions. Compared with the ultrasonic responses, its main envelope peaks are more concentrated in time and can be temporally associated with the UHF reference for subsequent pattern recognition and other signal analyses.

**Figure 18:**
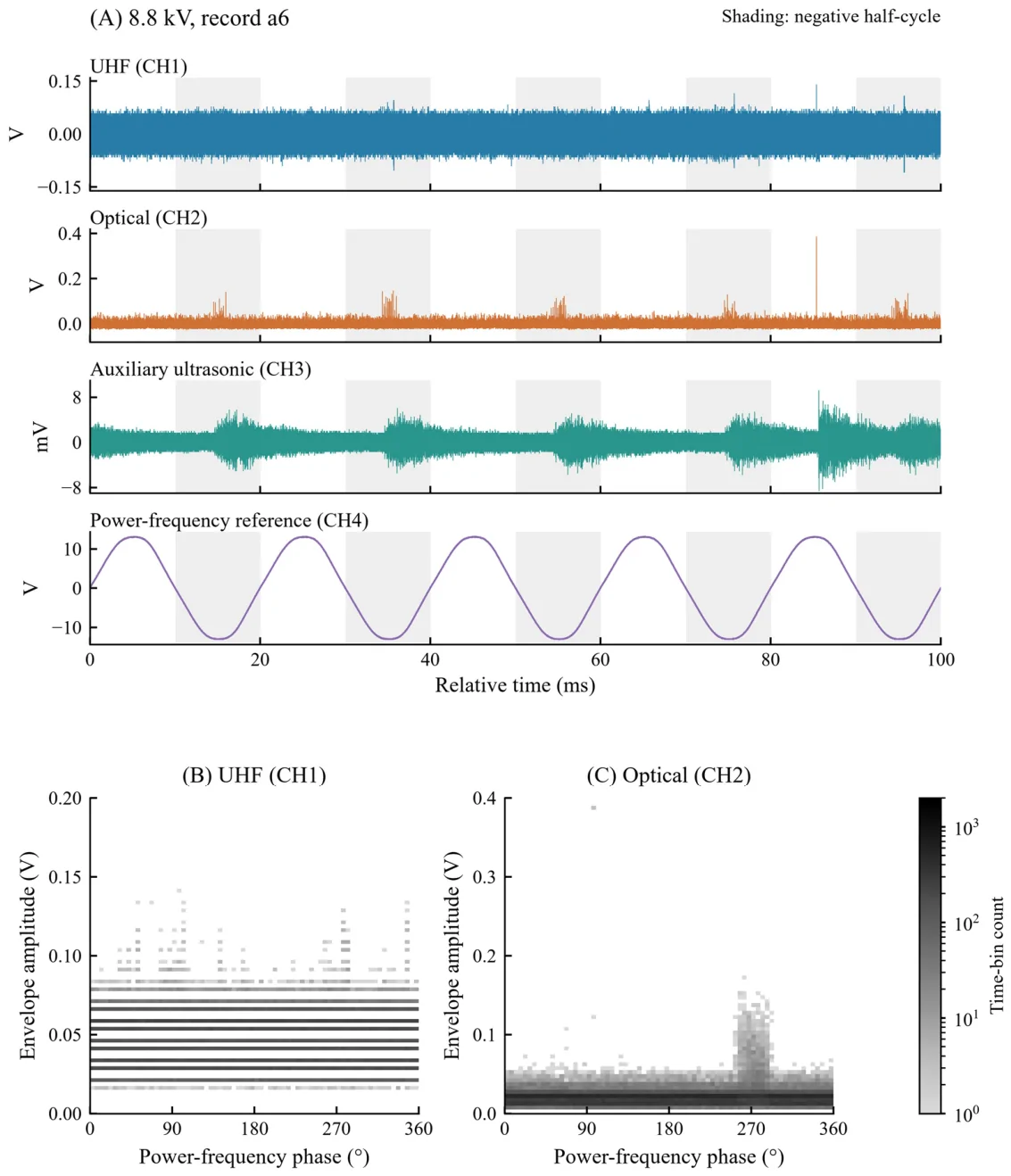
Responses at 8.8 kV: (**A**) Voltage extrema within 10 μs time bins for record a6; CH1–CH3 are centered by their median bin-midpoint baselines, and CH4 shows the power-frequency reference. Shading denotes negative half-cycles. (**B**) UHF and (**C**) optical envelope-amplitude distributions pooled separately from ten records, with 5° phase bins. The logarithmic color scale indicates time-bin counts, not independent pulse counts.

The 8.8 kV records provide a comparison at a weaker discharge stage under the same rod, defect, and mounting conditions. Figure 18A shows the voltage extrema for record a6, with the negative half-cycles identified from CH4. Optical clusters are visible during the negative half-cycle, with ultrasonic activity in nearby time intervals. Comparable clusters are not clearly resolved in the UHF trace, which is dominated by background fluctuations and sparse responses. The phase distributions pooled from the ten records in Figure 18B,C show the concentration of optical activity in the negative half-cycle. The 8.8 kV experimental records used to generate Figure 18 are available in the Supplementary Materials.

The two voltage levels reveal complementary optical information at different discharge stages. At 8.8 kV, negative-half-cycle optical clusters are more readily distinguished than the corresponding activity in the UHF records. At 12 kV, the main optical and UHF envelopes are temporally associated, while the optical channel also records lower-amplitude negative-half-cycle activity. These observations support the optical channel’s complementary response to weak activity under both tested voltage conditions. The ultrasonic channel provides supporting observations of activity in nearby intervals, without establishing one-to-one correspondence between individual optical and acoustic events.

Estimating received radiant power from the optical voltage requires calibration of the optical measurement chain at the acquisition settings used, accounting for its spectral and temporal response. Estimation of PD apparent charge additionally requires a conversion relationship validated against synchronous electrical measurements under specified discharge and propagation conditions. Such a relationship depends on the discharge characteristics, optical propagation path, and detector spectral response [19].

Gas composition and pressure can affect PD behavior and optical emission [5,19]. In full-scale GIS, internal conductors, insulating components, and shields can obstruct light or change its path through reflection and refraction at interfaces, altering the radiant flux and incident directions at the rod. The present tests did not cover the combined effects of operating gas, pressure, and internal GIS geometry. Further validation should compare the optimized and baseline structures in representative GIS configurations, using the insulating gas and filling conditions specified for the equipment. These comparisons should account for defect location and detector settings, with synchronous electrical measurements providing a discharge reference. Pressure resistance, gas tightness, and coupling stability of the feedthrough assembly also require assessment. The present optimization fixes the material and reference spectrum and does not incorporate differences between actual PD emission spectra into design selection. Further work could use a full-scale GIS test platform with representative PD defect models to measure and statistically characterize the emission spectra of different discharge types. Combining these measured spectra with the material’s spectral response and recorded optical signals would provide a basis for refining the taper profile and packaging for practical GIS conditions.

## 5. Conclusions

Rear-section design must account for propagation losses caused by metal contact and the finite aperture of the fiber entrance. Shortening the constant-diameter rear section reduces accumulated sidewall losses, while the selected parabolic taper improves the radiant flux received at the fiber entrance. With H = 8 mm and a retained gas gap, the mean received radiant flux across 27 source positions was 7.26 times that of the constant-diameter baseline. The minimum received flux, normalized by the mean of that baseline, was 0.710. Increasing the taper length to H = 20 mm raised the mean but reduced the normalized minimum to 0.355. Rear-section selection should therefore consider both the mean received flux and the flux at the least favorable source position.

In the integrating sphere tests, the parabolic sample provided the highest detector output under all 40 illumination conditions. Relative to the constant-diameter sample of equal total length, its output ratio under matching conditions had an equally weighted mean of 6.50, reflecting the combined gain from changes in taper profile and exit diameter. Relative to the linear taper with otherwise identical nominal dimensions, the mean ratio was 1.46, supporting the higher output of the parabolic profile under the tested conditions. Structural evaluation should therefore use detector output under common fiber reception conditions rather than relying solely on the light emerging from the entire rear end face.

At 8.8 kV, the optical channel recorded negative-half-cycle clusters with supporting ultrasonic activity in nearby intervals, while corresponding UHF clusters were difficult to distinguish from the background. In the 12 kV needle–plate corona tests in a laboratory GIS model containing air at 1 atm, the optical detection chain using this sample recorded responses temporally associated with the major UHF envelope and clusters of low-amplitude optical activity during the negative half-cycle, providing complementary information on the observed discharge activity. Together, the two voltage conditions show the optical channel’s complementary response to weak negative-half-cycle activity across the tested discharge stages. These discharge results apply to the tested needle–plate configuration at 8.8 and 12 kV in air at 1 atm. Evaluation of other defects and higher voltages should account for their emission spectra, propagation conditions, and measurement response range. The 10 μs extrema bins support envelope timing and 5° phase-interval statistics without resolving precise individual-pulse times or phases. Consistency across manufacturing batches requires further assessment.

## Figures and Tables

**Table 1:** Statistics of normalized radiant flux received at the rear end face for different front-section parameters.

L_1_ (mm)	H_1_ (mm)	Mean	Maximum	Minimum	Median
30	8.2	0.993	2.301	0.132	0.792
30	9	0.999	2.310	0.133	0.792
30	10	1.005	2.320	0.134	0.797
30	12	1.011	2.336	0.134	0.798
30	16	1.015	2.338	0.135	0.799
30	24	1.019	2.341	0.135	0.804
40	8.2	1.002	2.337	0.138	0.814
40	9	1.006	2.343	0.139	0.815
40	10	1.012	2.348	0.139	0.821
40	12	1.018	2.372	0.139	0.823
40	16	1.022	2.378	0.140	0.825
40	24	1.025	2.383	0.140	0.826
50	8.2	0.996	2.322	0.137	0.801
50	9	1.001	2.334	0.138	0.801
50	10	1.005	2.341	0.138	0.804
50	12	1.010	2.364	0.139	0.806
50	16	1.014	2.370	0.139	0.807
50	24	1.018	2.376	0.139	0.811
60	8.2	1.000	2.355	0.135	0.801
60	9	1.000	2.364	0.134	0.801
60	10	1.005	2.373	0.136	0.806
60	12	1.005	2.379	0.133	0.804
60	16	1.012	2.391	0.134	0.806
60	24	1.014	2.399	0.137	0.807

**Table 2:** Nominal geometric parameters of the four polymethyl methacrylate (PMMA) lightguide rod samples.

Parameter	S0	S1	S2	S3
Rear-section geometry	Long constant- diameter	Short constant- diameter	Parabolic taper	Linear taper
Body diameter D (mm)	8	8	8	8
Front-section length L_1_ (mm)	60	60	60	60
Rear-section length L_2_ (mm)	40	20	40	40
Total length L (mm)	100	80	100	100
Constant-diameter rear-section length (mm)	40	20	32	32
Taper length H (mm)	0	0	8	8
Rear end face diameter d (mm)	8	8	1	1
Number of samples	1	1	1	1

## Data Availability

The data supporting the findings of this study are provided as Supplementary Material.

## Supplementary Materials

The following supporting information can be downloaded at: https://www.mdpi.com/article/10.3390/s26216994/s1.

## Abbreviations

The following abbreviations are used in this manuscript:

GIS	Gas-insulated switchgear
LED	Light-emitting diode
PD	Partial discharge
PMMA	Polymethyl methacrylate
PMT	Photomultiplier tube
UHF	Ultrahigh frequency
